# Physicochemical Properties of α‑Pinene in Water Ice Analogs under Energetic Heavy-Ion Irradiation

**DOI:** 10.1021/acsearthspacechem.5c00152

**Published:** 2025-08-04

**Authors:** A. L. F. de Barros, D. V. Doreste, A. Ricca, Y. Murhej, E. F. da Silveira, P. Boduch, H. Rothard, A. Domaracka

**Affiliations:** † Departamento de Física, Centro Federal de Educação Tecnológica Celso Suckow da Fonseca, Av. Maracanã 229, Rio de Janeiro, Rio de Janeiro 20271-110, Brazil; ‡ Carl Sagan Center, 165047 SETI Institute, 339 Bernardo Ave, Suite 200, Mountain View, California 94043, United States; § NASA Ames Research Center, MS 245-6, Moffett Field, California 94035-1000, United States; ∥ Departamento de Física, Pontifícia Universidade Católica do Rio de Janeiro, Rua Marquês de São Vicente 225, Rio de Janeiro, Rio de Janeiro 22451-900, Brazil; ⊥ Centre de Recherche sur les Ions, les Matériaux et la Photonique Normandie University, ENSICAEN, UNICAEN, CEA, CNRS, CIMAP, Caen 14000, France

**Keywords:** astrochemistry, methods, laboratory, solid-state, astronomical instrumentation, methods, techniques, spectroscopic interstellar medium, nebulae, cosmic rays

## Abstract

Studying the physicochemical properties of ice in astronomical environments is crucial to understanding the chemical processes involved in cosmic events such as comet and planet formation. The physical characteristics and chemical evolution on the surfaces of cosmic objects such as comets or interstellar grains offer key insights into these processes. This study focuses on α-pinene, a carbon- and hydrogen-rich molecule, which serves as a model for investigating radical-driven synthesis of more complex molecules under space-like conditions. It also provides a useful analogy for complex terrestrial organic molecules and sheds light on how organic matter interacts with water and radiation in extraterrestrial environments. In this work, we simulate the effects of heavy-ion cosmic ray bombardment on chiral molecules in the interstellar medium by analyzing the radiolysis of a C_10_H_16_/H_2_O (1:1) mixture irradiated with 61.3 MeV ^84^Kr^15+^ ions. Fourier Transform Infrared (FTIR) spectroscopy is employed to monitor the chemical evolution of ice samples at 10 K, both before and after irradiation. We identify 12 C_*n*_H_*m*_ and ten C_*n*_H_*m*_O_*k*_ molecules, including complex products such as naphthalene (C_10_H_8_), glycolaldehyde (HCOCH_2_OH), and methyl formate (HCOOCH_3_). The most abundant hydrogenated product is acetylene (C_2_H_2_), followed by naphthalene (C_10_H_8_), while the most abundant oxygenated molecules are vinyl alcohol (CH_2_CHOH) and ethanol (CH_3_CH_2_OH). Notably, the formation of CO_2_ is minimal in this experiment. The destruction cross-sections of α-pinene and water in the (1:1) mixture are determined to be 3.5 and 6.4 × 10^–13^ cm^2^, respectively. The formation cross-sections for the products resulting from radiolysis are on average 2 × 10^–14^ cm^2^ for hydrocarbons and 0.6 × 10^–14^ cm^2^ for the oxygenated products.

## Graphical abstract

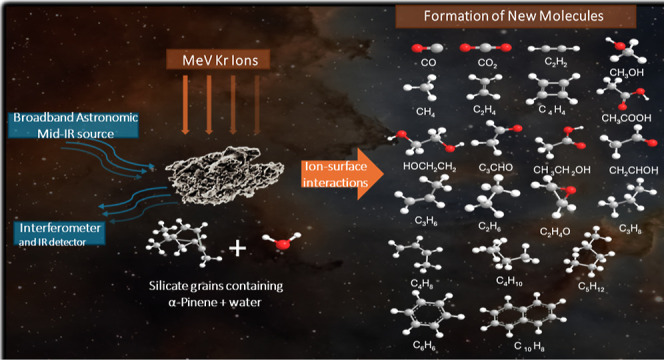

## Introduction

One of the most efficient methods for examining the structural and chemical modifications brought about by ion irradiation on frozen gases and materials is infrared spectroscopy. Studying the physicochemical properties of ice in astronomical environments is crucial to understanding the chemical processes involved in cosmic phenomena such as cometary activity, planetary surface evolution, and interstellar molecule formation. The surfaces of cosmic bodies, including comets, icy moons, and interstellar grains, are subject to energetic processing by cosmic rays and UV photons, which drive complex chemical evolution.^1–3^ Laboratory simulations using cryogenic ices irradiated with ions or photons provide essential insights into the stability and reactivity of organic compounds under these extreme conditions.^4,5^ These processes lead to the formation of complex organic molecules (COMs), many of which have been detected in the interstellar medium and on Solar System bodies.^6,7^

The formation of new molecular species that were not present in the original sample is one of the main consequences of ion irradiation, and new absorption features appear in the IR spectra. When hydrocarbons are in the original mixture, a refractory organic residue made up of OH, CH, CN, and CO groups is left over after the mixture warms up, and all volatile species are sublimated. There are several places in space where energetic processing, such as ion radiation, has a significant impact on the evolution of matter, such as planetary surfaces and molecular clouds in the interstellar medium. The chemical species present in these environments, rich in carbon, hydrogen, oxygen, and nitrogen, such as amino acids, proteins, and DNA molecules, are subject to intense investigation in several scientific fields.^8–11^

Understanding the origin of molecular asymmetry in prebiotic chemistry is essential as the chiral homogeneity of biopolymers is widely recognized as a prerequisite for self-replication and, consequently, for the emergence of life as we know it.^12,13^ Chirality is a universal feature of biological molecules: amino acids exhibit a predominance of left-handed (L) enantiomers, while sugars in nucleic acids are predominantly right-handed (D) enantiomers. The processes responsible for the initial symmetry breaking in the early solar system remain unresolved, with competing hypotheses invoking mechanisms such as circularly polarized light, asymmetric catalysis on mineral surfaces, and radiolytic interactions.^14,15^

In this context, biogenic volatile organic compounds (BVOCs), such as the chiral monoterpene α-pinene, serve as relevant molecular analogs for astrochemical investigations. Naturally emitted by terrestrial vegetation, α-pinene undergoes oxidation in earth’s atmosphere, contributing to aerosol formation. Its structural complexity and chirality make it an ideal candidate for studying the radiation-driven evolution of organic matter under astrophysical conditions. Moreover, similar terpenoid compounds may plausibly exist in planetary atmospheres, cometary nuclei, or icy grains in the interstellar medium (ISM), where they would be subjected to processing by ultraviolet photons and energetic particles.

Importantly, extraterrestrial chiral compounds preserved in comets and meteorites have endured exposure to ionizing radiation stemming from radionuclide decay and cosmic rays over billions of years.^16,17^ These high-energy processes can lead to racemization, degradation, or even the selective enrichment of one enantiomer. Therefore, investigating ion irradiation of α-pinene contributes to the understanding of the survivability and evolution of chiral molecules in space and chemical evolution in cold, radiation-rich environments. Such insights can be directly applied to prebiotic scenarios involving icy planetary surfaces, cometary ices, and interstellar dust grains.

The chiral bicyclic bridgehead of the α-pinene olefinic functional group makes it a desirable option for use as a monomer in the production of polymers. Numerous mechanistic approaches have been explored for the polymerization and functionalization of α-pinene, including free radical initiation, cationic polymerization, and metathesis catalysis. For example, α-pinene readily undergoes radical polymerization in the presence of thermal or photochemical initiators to form polyterpenes with varying molecular weights.^18^ Cationic polymerization, often using Lewis or Brønsted acids, has been widely studied due to its capacity to generate regioselective and stereospecific products.^19^ More recently, α-pinene derivatives have also been used in metathesis reactions catalyzed by ruthenium complexes to produce functionalized macromolecules and oligomers.^20^

Chirality is not preserved when high-energy radiation interacts with primitive chiral compounds.^21,22^ Cosmic rays acting on the surfaces of comets and meteorites are responsible for most of the radiation processing on them; however, at a depth of 20 m, the cosmic rays are completely attenuated, and radionuclide decay is the only local source of radiation.^21,22^ Cataldo^22^ has confirmed that interactions between primitive chiral substances and high-energy radiation do not sustain chirality.

Recently, de Barros et al.^23^ and Ramachandran et al.^24^ have studied pure α-pinene (C_10_H_16_) at 10 K irradiated with 61.3 MeV ^84^Kr^15+^ beams and vacuum ultraviolet (VUV) photoabsorption, respectively. In both experiments, the spectra of pure α-pinene show similarities between the ice and the gas phases. In the present study, we observed new species not detected in the pure α-pinene case: most notably, small oxygenated molecules.

The krypton ion was selected as the projectile in this study due to its high atomic mass and charge state, which make it an effective analog for galactic cosmic rays (GCRs) interacting with icy surfaces in space. Krypton ions with MeV energies deposit their energy into the target predominantly through electronic stopping (i.e., inelastic interactions with target electrons), leading to dense energy tracks similar to those caused by high-energy heavy ions in astrophysical environments.

It is well-known that many complex organic molecules (COMs) have been observed in the interstellar medium (ISM), and their formation has been investigated in laboratory experiments.^25–28^ In astrophysical settings, chemical analysis is complicated by the fact that many ice species are mixed. Thus, anticipating the result of ice irradiation in space requires a quantitative understanding of how different mixture constituents affect the ice radiolysis chemistry.^1,3,29^ Due to its prominence and its known effects on the ice-binding environment, this study focuses on the effect of H_2_O ice during heavy-ion irradiation with a C_10_H_16_/H_2_O (1:1) ice mixture. It is not expected that this mixture matches exactly the multicomponent ice mixture found in space. Instead, we are using it to study the basic properties of ice astrochemistry.

Water ice is ubiquitous in space and is observed in a variety of astrophysical environments, including dense molecular clouds, comets, and icy planetary bodies.^30,31^ It remains unclear which phase of ice, amorphous or crystalline, most accurately represents water ice on the surfaces of interstellar dust grains or on the planetary and lunar regolith. Laboratory and observational studies suggest that amorphous ice likely dominates under typical interstellar conditions due to its low-temperature formation, although structural transitions to crystalline forms may occur upon thermal or energetic processing.^32–35^

Water is a very complex ice, with 17 distinct phases that are either crystalline or amorphous.^31^ Observations show that interstellar icy grain mantles are dominated by H_2_O, which is typically the most abundant component by a factor of about 2:1 compared to the next most common species. These include CO (∼20 to 30%), CO_2_ (∼15 to 25%), CH_3_OH (∼5 to 30%), NH_3_ (∼5 to 10%), CH_4_ (∼2 to 10%), and H_2_CO (a few percent).^36–39^ The relative abundances of these minor components vary depending on the specific environment probed along the line of sight. In radiolysis of α-pinene–water mixtures, H_2_O influences radical mobility and trapping: in water-rich ices, binding energies are generally higher than those in pure ices, which alters diffusion rates and reaction probabilities. Water can also physically trap radicals at low temperatures, modifying the resulting chemistry.^40,41^

The mixture of α-pinene with H_2_O affects astrochemistry in ways other than by simply increasing the level of OH radicals in the ice. According to Collings et al.,^40^ the binding energies in H_2_O-rich ices are generally different from those in pure ices, which may have an impact on radical diffusion. At low temperatures, radicals can also physically get trapped in water ice, as is often seen with volatile compounds.^41^ Different radicals are likely to exhibit stronger or weaker interactions with water, which could lead to unexpected changes in the chemistry. We examine the relative significance of these possible H_2_O impacts on the radiochemistry of C_*n*_H_*m*_.

For the first time, a mixture of α-pinene and H_2_O (1:1) at 10 K was irradiated with heavy ions, and mid-infrared (MIR) spectroscopy (6500–450 cm^–1^ region) was used to detect the evolution of the reactants and radiolysis products as a function of the irradiation fluence, to obtain their formation and destruction cross-sections, and to quantify the band strengths.

## Experimental Procedures

The investigations were carried out using the IGLIAS (French acronym for “Irradiation of astrophysical ices”) setup at CIMAP-CIRIL on the IRRadiation SUD beamline of the French National Heavy Ion Accelerator (GANIL).^42^ The IRRSUD beamline routinely provides heavy-ion beams such as ^84^Kr^15+^ with precise control over the energy and fluence. It provides a well-characterized, reproducible, and controlled method to simulate the chemical effects of high-LET (linear energy transfer) cosmic rays, particularly those from heavy nuclei like Fe, allowing laboratory simulations of space radiation environments like organic matter embedded in ices. The energy deposition at the microscopic level is similar for heavy ions such as Fe, Ni, and Kr.^43,44^ Fourier Transform Infrared (FTIR) spectroscopy was used in the transmission mode covering the 4000–600 cm^–1^ range, with a spectral resolution of 1 cm^–1^ and an average of 70 scans. All infrared spectra were corrected using background subtraction. Prior to each deposition, a background spectrum was recorded with the clean substrate under the same vacuum and temperature conditions to maintain spectral consistency.^23^ The spectra in the mid-IR region were collected to track the development of column densities as a function of the beam fluence.

The mixture of α-pinene–water vapor was deposited onto a ZnSe optical flat crystal window in an ultrahigh vacuum chamber (base pressure <2 × 10^–10^ mbar). The substrate was a 2 mm thick, 20 mm diameter optical-grade window with a polished surface selected for its excellent transmission in the mid-infrared range. Both components, α-pinene vapor (Sigma-Aldrich, purity better than 98%) and Milli-Q water vapor (purified through multiple freeze–pump–thaw cycles), were premixed in the gas ramp and simultaneously admitted into the chamber via a deposition tube placed 15 mm in front of the IR window at controlled partial pressures to form a uniform ice film at 10 K. For more details, see Augé et al.^42^ Homogeneity of the film was confirmed by the reproducibility of infrared spectra in terms of band positions, shapes, and integrated absorbances across repeated experiments, indicating consistent mixing and uniform coverage of the substrate.

A commercially available α-pinene sample (98.8% from Sigma-Aldrich) was purified before using a series of freeze–pump–thaw cycles to remove air and other volatile contaminants at the liquid N_2_ temperature. Ultrapure liquid water with a resistivity of 18.2 MΩ cm was purged with liquid nitrogen, and α-pinene vapors were then added to a prechamber that had been evacuated to a pressure of 10^–6^ mbar. This value was used to verify the quality of the water and to ensure minimal contamination during sample preparation, which is especially important for astrochemical experiments where trace impurities could significantly alter radiolysis outcomes. The α-pinene vapor was fed into the main chamber at a pressure of 2 × 10^–7^ mbar after the sample holder had cooled. The remaining gas pressures for the IGLIAS setup were within the ranges of 10^–8^ and 10^–10^ during ion irradiation.

The C_10_H_16_/H_2_O (1:1) ice film was irradiated at normal incidence with 61.3 MeV ^84^Kr^15+^ projectiles. According to the SRIM code,^45^ the energy transfer rate per projectile through electronic interaction was approximately *S*_e_ = 5.80 × 10^3^ keV μm^–1^ which is sufficient to initiate radiolytic reactions that form and destroy complex molecules. The range of these projectiles was 10 μm, which is large enough to completely traverse the 3.4 μm thick ice layer.

The thickness, η, of the α-pinene and water was determined by eq 1 in microns

1 $$\eta =\frac{N_{0}M10^{4}}{\rho N_{A}}$$

Here, *N*_0_ represents the initial column density of the ice, where *N*_0_(C_10_H_16_) = 8.98 and *N*_0_(H_2_O) = 11.1 × 10^18^ molecules cm^–2^, *M* is the total molecular mass, and *N*_A_ is Avogadro’s constant. The density ρ used for α-pinene was 0.858 g cm^–3^,^46^ and for water, it was 1.1 g cm^–3^.^47^

## Results and Discussion

### Comparison of the IR Spectra of Unirradiated Pure α-Pinene and α-Pinene in Water Ices

The 4000 to 650 cm^–1^ FTIR spectra of pure nonirradiated α-pinene and the water mixture are shown in Figure 1 and are presented in three subregions for clarity. The spectra of pure α-pinene and those of the mixture with water show good correlations with each other (Figure 1). Three water bands were observed around ∼760 cm^–1^ as a ν_R_ libration, ∼1650 cm^–1^ as a ν_2_ bend, and at ∼3280 cm^–1^ as a ν_3_.^47^ Most of the α-pinene bands were observed in the mixture spectra, and the shifts in the band positions are listed in Table 1.

**Figure 1:**
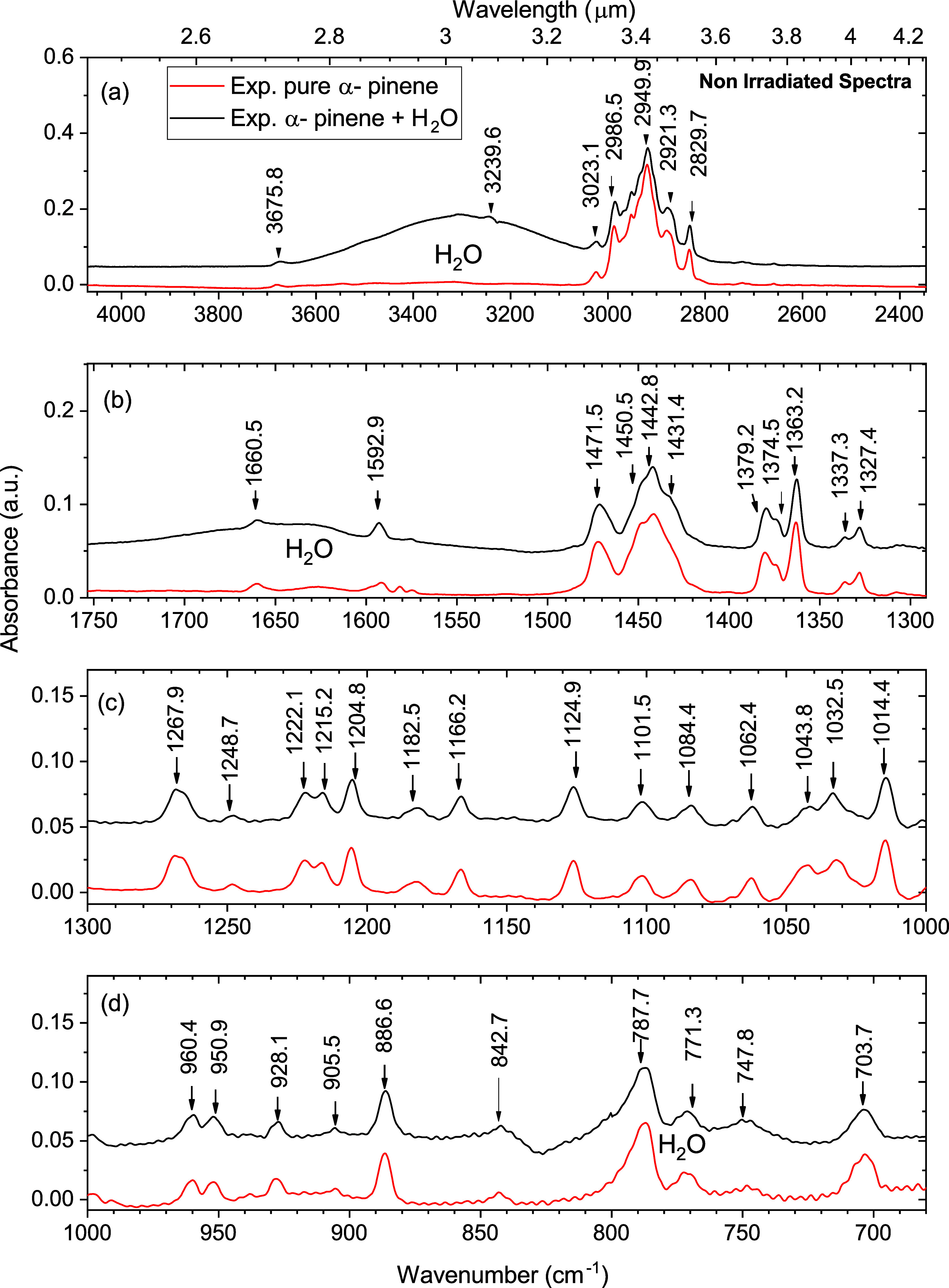
Comparison of mid-infrared spectra of nonirradiated samples of pure α-pinene (bottom—red) and the mixture C_10_H_16_/H_2_O (1:1) (top—black) at 10 K. (a) Spectral region from 4000 to 2000 cm^–1^; (b) 1750 to 1300 cm^–1^; (c) 1300 to 1000 cm^–1^; and (d) 1000 to 650 cm^–1^.

**Table 1:** Summary of Neutral C_10_H_16_/H_2_O (1:1) Mixture and Pure α-Pinene (C_10_H_16_) Band Positions in the MIR (600 to 4000 cm^–1^) Region (in cm^–1^), *A*-Values (km mol^–1^), and Band Assignments

(C_10_H_16_/H_2_O) (1:1)	C_10_H_16_ pure^a^	Integrated region	*A* value^a^ (km mol^–1^)	Assignment/Comments
703.8	703.7	713.9–696.6	2.26	ring deformation
747.8	747.8	779.1–762.7	2.76	ring deformation
760.2		750.2–765.3		H_2_O ν_R_ libration^b^
771.3	770.0	807.5–779.1	2.77	ring deformation
787.7	787.5	806.6–801.7	12.3	C–H bending
842.7	842.1	854.8–836.5	1.18	C skeleton deformation; C–H bending
886.6	884.4	894.8–878.4	4.87	C skeleton deformation; C–H bending
905.5	904.4	911.2–902.5	0.45	C skeleton deformation
928.1	927.5	935.3–923.2	1.35	C skeleton deformation; CH_3_ asymmetric deformation
950.9	949.7	656.5–944.5	1.26	C skeleton deformation; CH_2_ twisting; CH_3_ asymmetric deformation
960.4	960.0	968.6–956.0	1.61	CH_3_ asymmetric deformation
997.6	998.1	1010.9–1011.6	0.16	CH_3_ asymmetric deformation
1014.4	1014.2	1022.1–1006.6	4.43	CH_3_ asymmetric deformation; C–H bending; CH_2_ twisting
1032.5	1032.8	1038.9–1022.5	2.01	CH_2_ twisting; C–H bending
1043.8	1041.9	1052.9–1038.5	1.96	CH_2_ twisting; C–H bending
1062.4	1061.9	1068.8–1056.3	2.16	CH_2_ twisting; C–H bending
1084.4	1083.8	1093.4–1077.0	2.37	CH_2_ twisting; C–H bending
1101.5	1101.0	1110.3–1093.9	2.41	CH bending; CH_2_ twisting
1124.9	1125.9	1134.9–1118.0	3.53	CH bending
1066.2	1166.5	1172.9–1159.9	2.12	CH bending; C–C_5_C bending; CH_2_ twisting
1182.5	1182.8	1190.8–1175.9	1.33	C–C–C bending; CH bending; CH_3_ asymmetric deformation
1204.8	1205.5	1210.6–1198.5	2.95	CH_2_ wagging; CH_2_ twisting; CH_3_ asymmetric deformation
1215.2	1215.5	1215.9–1210.1	1.53	CH_2_ wagging; C–H bending; CH_2_ twisting
1222.1	1222.4	1230.3–1222.6	2.56	CH_2_ wagging; C–H bending; CH_2_ twisting
1248.7	1248.7	1254.9–1242.4	0.53	C–H bending
1267.9	1268.3	1280.9–1254.9	5.01	C–H bending; CH_2_ twisting
1308.1	1307.3	1314.2–1297.4	0.51	C–H bending; CH_2_ wagging
1327.4	1327.4	1333.5–1322.4	1.57	C–C stretching; C–H bending; CH_2_ wagging
1337.3	1337.9	1341.7–1333.0	0.44	CH_2_ wagging; C–H bending
1363.2	1363.0	1370.1–1354.7	7.94	CH_3_ symmetric deformation
1374.5	1373.9	1385.6–1370.1	1.77	CH_3_ symmetric deformation
1379.2	1381.1	1389.4–1370.1	5.08	CH_3_ symmetric deformation
1431.4	1431.7	1460.8–1409.7	4.02	CH_2_ scissoring
1442.8	1441.5	1460.8–1409.7	4.01	CH_2_ scissoring
1450.5	1449.2	1504.2–1463.2	15.0	CH_3_ asymmetric deformation
1471.5	1471.5	1481.9–1460.8	11.3	CH_2_ scissoring; CH_3_ asymmetric deformation
1582.1	1581.2	1585.6–1577.9	0.39	
1592.9	1593.4	1605.9–1585.6	1.66	
1645.7		1652.3–1610.8		H_2_O ν_2_ bending^b^
1660.5	1661.2	1657.2–1674.5	0.09	C=C stretching
2829.7	2832.3	2825.3–2850.4	0.18	CH_2_ symmetric stretching pos. 4
2921.3	2917.6	2915.2–2928.2	0.42	bridging C–H (vinyl) stretching
2949.9	2949.9	2943.8–2955.3	0.27	CH_3_ asymmetric stretching pos. 2
2986.5	2989.5	2982.6–2993.5	0.26	CH_2_ asymmetric stretching pos. 7
3023.1	3023.1	3021.7–3028.9	0.14	C–H vinyl stretching
3309.1		3632.4–3060.5		H_2_O ν_3_ asymmetric stretching^b^
3675.8		3670.5–3660.2		OH dangling bonds (db) stretching^b^

a Note: *A*-values ≥ 2 km mol^–1^ are given in de Barros et al.^23^ for pure α-pinene.

b The band position of water bands (see Bouilloud et al.^47^).

The effect of water ice on the vibrational bands of α-pinene, as shown in Table 1, can be discussed by examining the shifts and appearances of new bands upon mixing with water. The presence of water molecules alters the vibrational modes of the α-pinene molecules, primarily by engaging in hydrogen bonding or by inducing new interaction pathways that affect the molecular vibrations of both components, such as

(i) In the region of C–H bending and C–C stretching, several bands shift in the presence of water. For example, 1450.5 cm^–1^ and 1471.5 cm^–1^ exhibit deformation modes that likely involve water-induced perturbations of the α-pinene structure. Specifically, the band near 1471 cm^–1^, typically associated with asymmetric deformation of the methylene group (−CH_2_−) and vinyl C–H bending modes, is sensitive to changes in the molecular environment. The presence of water can influence the vibrational coupling of these modes through dipole–dipole interactions or by stabilizing conformers that are otherwise unfavorable in the pure phase.

Therefore, the combined influence of α-pinene–water hydrogen bonding and potential substrate interactions provides a plausible explanation for the shifts and intensity variations observed near 1471 cm^–1^. These effects are consistent with previous studies of terpene and hydrocarbon mixtures on cold surfaces under vacuum.^1,48^

(ii) Certain bands in the bending region of CH, such as those at 787.7 cm^–1^ and 842.7 cm^–1^, show changes in intensity when mixed with water. The increase in intensity of some bands, such as 787.7 cm^–1^, could be due to the direct interaction between water molecules and the hydrophobic groups of α-pinene, modifying the distribution of electron density and altering vibrational characteristics.
(iii) The broadening of peaks, especially in the C–H stretching region (e.g., 2921.3 cm^–1^ and 2949.9 cm^–1^), indicates that water can cause additional broadening due to interactions between α-pinene and water molecules. The CH_2_ asymmetric stretching mode at 2986.5 cm^–1^ also exhibits some broadening, further supporting the idea that water interacts with the α-pinene structure, leading to more complex vibrational behavior.

### Radiolysis of α-Pinene in Water Ices

A comparison of the spectra of irradiated (green bottom spectra; fluence of 2 × 10^12^ ions cm^–2^) and nonirradiated (top in black) water mixtures is presented in Figure 2 and shows that new bands appear in the infrared spectrum during the irradiation process up to the final fluence of 2 × 10^–12^ ions cm^–2^. Plausible assignments of these new bands are shown in Figure 2, and the list of observed new species formed, together with their corresponding vibrational assignments, is given in Tables 2 and 3. A total of 12 new hydrocarbons were observed during the 61.3 MeV ^84^Kr^15+^ ion bombardment, namely, methane (CH_4_), acetylene (C_2_H_2_), ethylene (C_2_H_4_), propylene (C_3_H_6_), propane (C_3_H_8_), *n*-butane (C_4_H_10_), butene (C_4_H_8_), benzene (C_6_H_6_), ethane (C_2_H_6_), vinylacetylene (C_4_H_4_), naphthalene (C_10_H_8_), pentane (C_5_H_12_), and 2-methyl-1,3-butadiene or isoprene (C_5_H_8_). We did not observe evident signs of racemization.^21,22^

**Figure 2:**
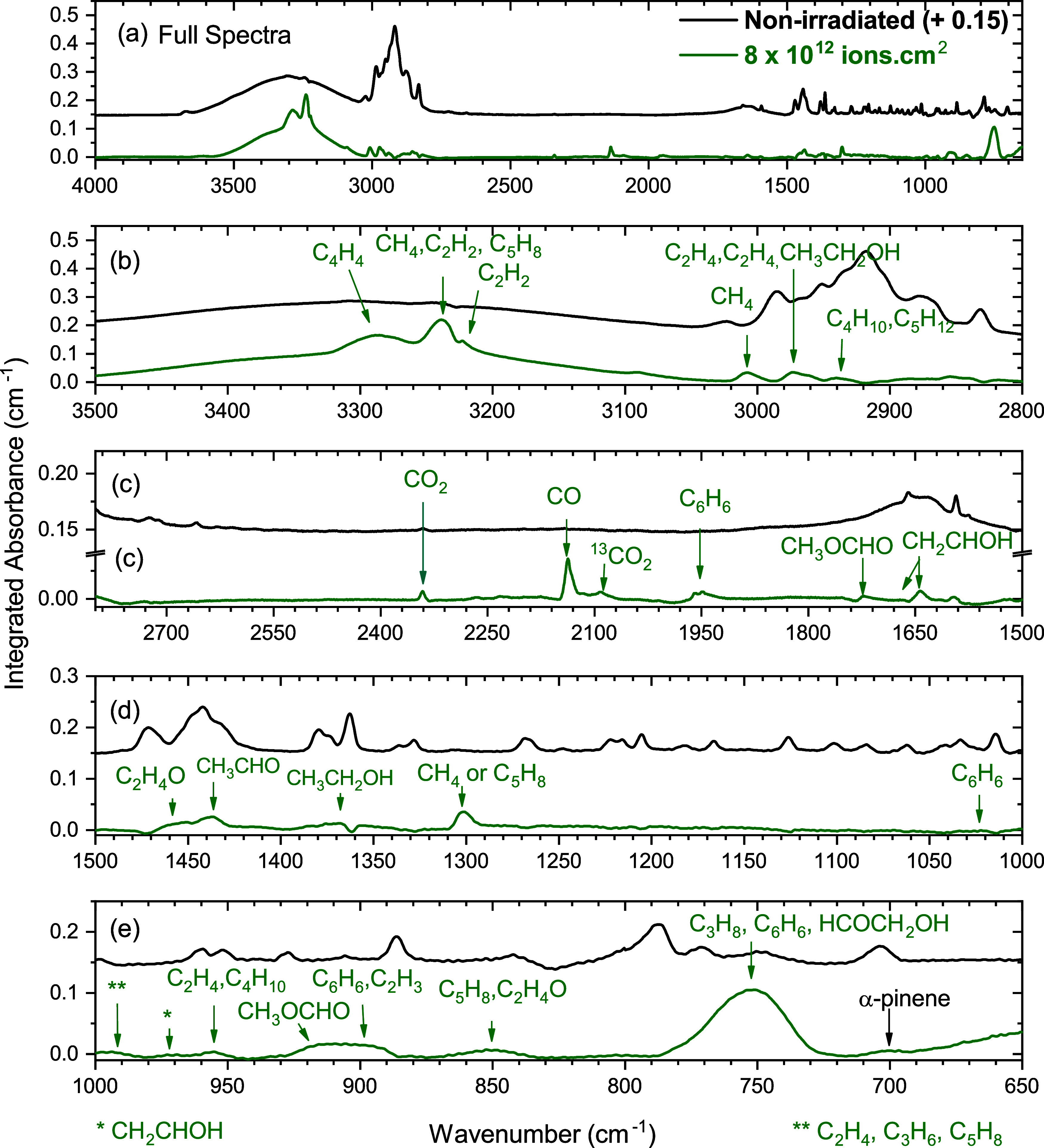
Comparison of the spectra of nonirradiated (top in black) and irradiated C_10_H_16_/H_2_O (1:1) at 10 K using a fluence of 8 × 10^12^ ions cm^–2^ (bottom in green). (a) Full spectra from 4000 to 650 cm^–1^; (b) from 3500 to 2800 cm^–1^; (c) from 2800 to 1500 cm^–1^; (d) from 1500 to 1000 cm^–1^; and (e) from 1000 to 650 cm^–1^.

**Table 2:** Wavelengths and Absorption Modes (in cm^–1^) Observed during Irradiation at a Fluence of 2 × 10^12^ Ions cm^–2^ of C_10_H_16_/H_2_O (1:1) Ice in the Region from 650 to 1470 cm^–1^

Position C_10_H_16_/H_2_O (1:1)	Literature values	Identified molecules	Absorption mode
655.5	660	CO_2_ (carbon dioxide)^43,47,49^	ν_2_ bending
752.4	750	C_3_H_8_ (propane)^44,50^	ν_5_ CH_2_, CH_3_ twisting, rocking
	752	HCOCH_2_OH (glycolaldehyde)^51,52^	OCC + (CC,COH) scissoring
	754	C_6_H_6_ (benzene)^10^	C–H out-of-plane bending
758.2	758	CH_3_CHO (acetaldehyde)^53^	ν_14_ CH_3_ twisting
	761	C_2_H_2_ (acetylene)^10,54^	ν_5_ C≡C–H bending
	760	C_5_H_8_ (propane)^48^	CH_2_ twisting
868.0	868	*c*-C_2_H_4_O (ethylene oxide)^53^	ν_5_ ring deformation
	865	HOCH_2_CH_2_OH (ethylene glycol)^51^	ν_6_ CH_2_ + CH_2_ rocking
	866	HCOCH_2_OH (glycolaldehyde)^52,55^	ν_7_ C–H bend and O–H deform
908.8	909	C_6_H_6_ (benzene)^56^	ν_18_ C–H out-of-plane bending
	910	C_4_H_8_ (butene)^54^	=CH_2_ wagging
917.2	916	CH_3_OCHO (methyl formate)^51^	ν_5_ CO stretching
	916	C_2_H_2_ (acetylene)^56^	C≡C–H
954.2	952	C_2_H_4_ (ethylene)^54^	ν_7_ CH_2_wagging
	953	C_5_H_8_ (2-methyl-1,3-butadiene or isoprene)^48^	CH_2_ wagging/CH_2_ twisting
958.7	960	C_4_H_10_ (*n*-butane)^54^	ν_16_ CH_3_ rocking
	957	CH_3_COOH (acetic acid)^57^	ν_9_ CH_3_ rocking
994.5	993	C_3_H_6_ (propylene)^47^	CH bending
	993	C_4_H_8_ (butene)^54^	HC=CH wagging
	993	C_5_H_8_ (2-methyl-1,3-butadiene or isoprene)^48^	CH bending/CH_2_ wagging/CH_2_ twisting
1023.7	1024	C_10_H_8_ (naphthalene)^58^	C–H bending
1032.3	1029	CH_3_CH_2_OH (ethanol)^51^	ν_6_ OCC *asym*. stretching
	1031	CH_3_OH (methanol)^47,53^	ν_8_ CO stretching
1037.5	1038	C_6_H_6_ (benzene)^10,56^	ν_18_ C–H in-plane bending
	1037	HOCH_2_CH_2_OH (ethylene glycol)^51,57^	ν_8_ OCC *sym*. stretching
1067.1	1070	HCOCH_2_OH (glycolaldehyde)^51^	ν_7_ CO stretching
	1066	HOCH_2_CH_2_OH (ethylene glycol)^51^	ν_10_ CH_2_ rock + twist + O–H rock
1090.1	1093	CH_3_CH_2_OH (ethanol)^55^	O–H or C–H bending
	1090	HOCH_2_CH_2_OH (ethylene glycol)^51^	ν_9_ C–O stretching
1301.7	1304	CH_4_ (methane)^44,47,54^	ν_4_ C–H bending
	1293	C_5_H_8_ (propane)	CH bending
1387.2	1390	C_10_H_8_ (naphthalene)^58^	C–C stretching and C–H bending
	1380	C_5_H_12_ (pentane)^59^	δ_s_-CH_3_
1432.2	1431	CH_3_CHO (acetaldehyde)^53^	ν_5_ CH_3_ deform
1464.7	1465	*c*-C_2_H_4_O (ethylene oxide)^53^	ν_2_ CH_2_ scissoring
	1462	C_10_H_8_ (naphthalene)^58^	O–C–H bending or C–H wagging
	1460	C_5_H_12_ (pentane)^59^	δ_as_-CH_3_ deformation

**Table 3:** Wavelengths and Absorption Modes Observed during Irradiation of C_10_H_16_/H_2_O (1:1) Ice at a Fluence of 2 × 10^12^ Ions cm^–2^ in the Region from 1470 to 3610 cm^–1^

Position C_10_H_16_/H_2_O (1:1)	Literature values	Identified molecules	Absorption mode
1473.9	1475	*c*-C_2_H_4_O (ethylene oxide)^53^	CH_2_ scissoring
	1475	CH_3_CH_2_OH (ethanol)^51^	ν_15_ (CH_2_ + CH_2_ of CH_3_) scissoring
	1469	HOCH_2_CH_2_OH (ethylene glycol)^51^	ν_17_ (CH_2_ + CH_2_) scissoring
1596.3	1599	C_4_H_4_ (vinylacetylene)^60^	ν_6_ C=C stretching
	1597	C_10_H_8_ (naphthalene)^58^	C–H bending or C–H out-of-plane
1670.1	1669	*cis*-CH_2_CHOH (vinyl alcohol)^53^	CC stretching
	1671.9	C_10_H_8_ (naphthalene)^58^	C–H bending or C–H out-of-plane
1642.8	1639	*cis*-CH_2_CHOH (vinyl alcohol)^53^	ν_5_ CC stretching
	1643	C_4_H_8_ (butene)^54^	C=C stretching
		C_3_H_6_ (propylene)^50^	C=C stretching
1722.8	1723	CH_3_CHO (acetaldehyde)^53^	ν_4_ CO stretching
	1717	CH_3_OCHO (methyl formate)^51,61^	ν_14_ CO stretching
	1723	CH_3_COOH (acetic acid)^57^	ν_4_ CO stretching
1732.0	1733	C_10_H_8_ (naphthalene)^58^	C–H bending
	1731	CH_3_CH_2_OH (ethanol)^51,55^	ν_12_ (CH_3_ + CH_2_) wagging
	1731	CH_3_OCHO (methyl formate)^51,61^	ν_4_ CO stretching
1950.7	1950	C_2_H_2_ (acetylene)^44^	CC C≡C
1960.3	1960	C_6_H_6_ (benzene)^10,56^	ν_17_ + ν_5_ C–H bend and ring deform
	1961	C_2_H_2_ (acetylene)^62^	ν_2_ C≡C stretching
	1959	C_10_H_8_ (naphthalene)^58^	C–H bending or ring deformation
2091.6	2092	^13^CO (carbon monoxide)^43,47,63^	ν_1_ stretching ^13^CO
2137.2	2138	CO (carbon monoxide)^43,49,53^	ν_1_ stretching ^12^CO
2344.5	2346	CO_2_ (carbon dioxide)^43,47,49^	ν_3_ CO_2_ stretching
2932.6	2930	C_5_H_12_ (pentane)^59^	CH_2_ *sym*. stretching
	2930	C_4_H_10_ (*n*-butene)^54^	ν_13_ CH_2_ *sym*. stretching
	2927	CH_3_CH_2_OH (ethanol)^51^	ν_17_ CH_3_ *sym*. stretching
2973.8	2973	C_4_H_4_ (vinylacetylene)^60^	ν_6_ + ν_7_ C–H stretch or C–H deform
	2974	CH_3_CH_2_OH (ethanol)^51^	ν_18_ CH_2_ of CH_3_ *sym*. stretching
	2974	C_2_H_4_ (ethylene)^5^	CH_2_ *sym*. Stretching
	2975	C_2_H_6_ (ethane)^54,64^	ν_10_ CH_3_ stretching
3009.2	3009	CH_4_ (methane)^44,47,65^	ν_3_ C–H stretching
	3008	CH_3_CH_2_OH (ethanol)^51^	ν_20_ CH of CH_3_ stretching
3228.7	3232	C_2_H_2_ (acetylene)^10^	C≡C–H *asym*. stretching
3239.4	3237	C_2_H_2_ (acetylene)^10,44,62^	ν_3_ C–H stretching
	3240	CH_4_ (methane)^44^	ν_3_ C–H stretching
3288.1	3287	C_4_H_4_ (vinylacetylene)^54,60^	ν_1_ C–H stretching
	3288	C_2_H_2_ (acetylene)^56^	aggregates
3609.2	3600	CO_2_ (carbon dioxide)^43,47,49^	2ν_2_ + ν_3_ CO_2_ bending

Due to the presence of water in the mixture, the formation of oxygenated products such as carbon monoxide (CO) and (^13^CO), carbon dioxide (CO_2_), acetaldehyde (CH_3_CHO), ethanol (CH_3_CH_2_OH), ethylene oxide (C_2_H_4_O), vinyl alcohol (CH_2_CHOH), glycolaldehyde (HCOCH_2_OH), methyl formate (CH_3_OCHO), and ethylene glycol (HOCH_2_CH_2_OH) was also observed.

The evolution of the spectra of irradiated C_10_H_16_/H_2_O (1:1) at 10 K as a function of fluence is shown in Figure 3. It is important to note that some important methanol bands, such as 1011 cm^–1^ ν_8_ (^13^C), 1041 cm^–1^ ν_8_, 1157 cm^–1^ ν_7_, 1188 cm^–1^ 2ν_12_, 1428 cm^–1^ ν_6_, and 1444 cm^–1^ ν_5_ were observed by Bennett et al.^51^ Such bands are close to the α-pinene bands (see Table 1); therefore, for methanol, we use the band located at 1032.3 cm^–1^ in the current analysis.

**Figure 3:**
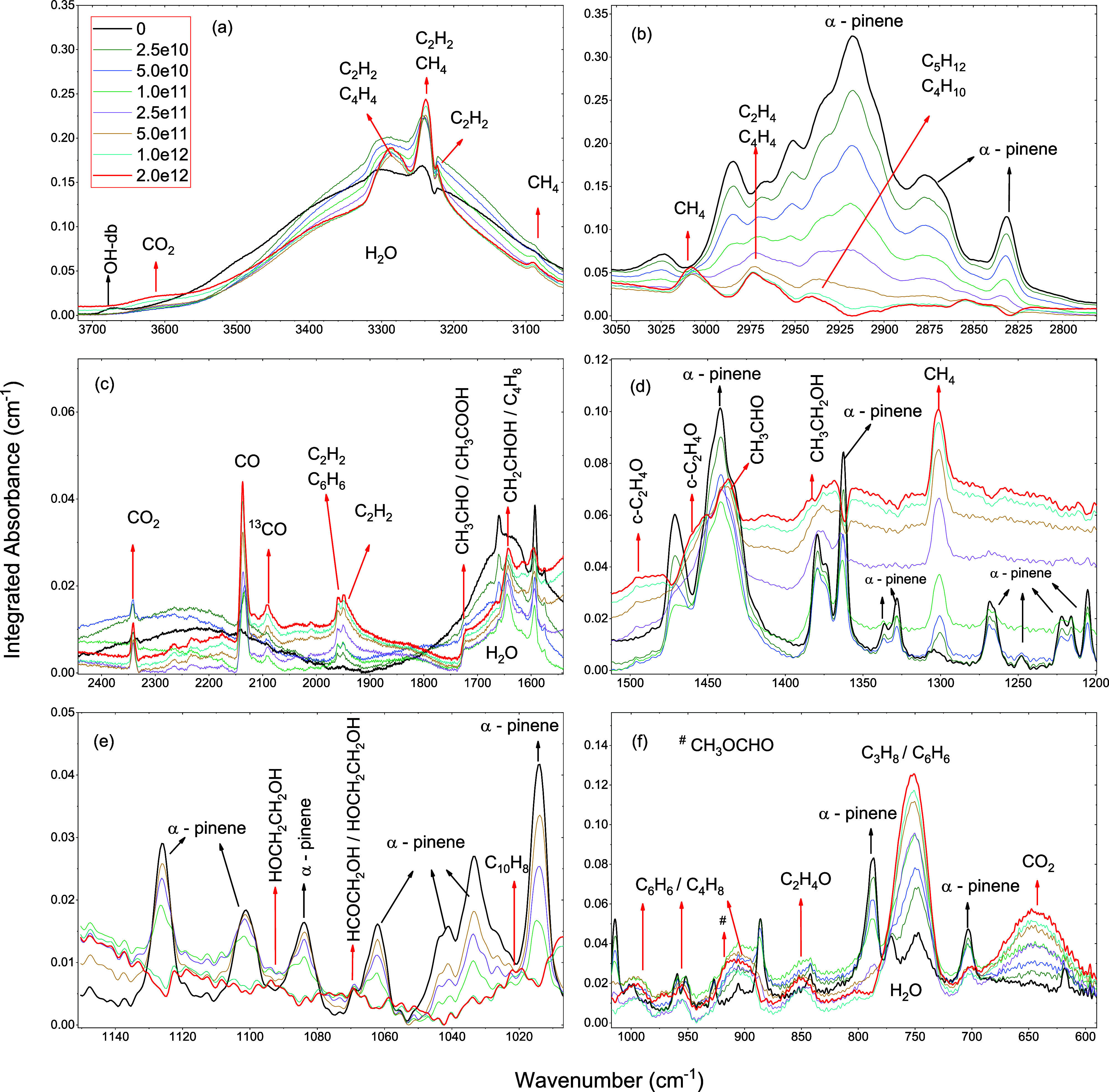
Spectra of irradiated C_10_H_16_/H_2_O (1:1) at 10 K as a function of fluence. (a) Spectral region from 3700 to 3050 cm^–1^; (b) from 3050 to 2790 cm^–1^; (c) from 2430 to 1550 cm^–1^; (d) from 1500 to 1200 cm^–1^; (e) from 1150 to 1010 cm^–1^; and (f) from 1000 to 600 cm^–1^.

As seen in Figure 3, as the absorbance of the molecular species during irradiation increases, some infrared vibrational bands begin to overlap. Tables 2 and 3 reveal several bands associated with more than one molecular species. This clearly demonstrates that hydrocarbons heavier than C_2_H_*m*_ exhibit a significant overlap of their most intense vibrational bands, considerably complicating their identification. In accordance with this sequence, let us now discuss each product in detail:

Two fundamental vibration bands of methane are observed in the mixture: 1301.7 (ν_4_ C–H bending) and 3009.2 cm^–1^ (ν_3_ C–H bending),^44,47,49,65^ as can be seen in Figure 3b,d. Six acetylene bands are identified at 758.2 (ν_5_ C≡C−H bending), 917.2 (C≡C-H), 1950.7 (C≡C), 1960.3 (ν2 C≡C stretching), 3228.7 cm^−1^ (C≡C−H *asym*. str.), and 3288.1 cm^−1^ (aggregates) in good agreement with experiments^44,51,54,56,57,66^ (details in Figure 3a,c). The ethylene bands are identified at approximately 954.2 cm^–1^ (ν_7_ CH_2_ wagging) and 2973.8 cm^–1^ (CH_2_ *sym*. stretch) according to the values in the literature.^49,54,65^ Two propylene bands are identified as CH bend at 994.5 cm^–1^ and C=C stretch at 1642.8 cm^–1^,^50^ and two propane bands are observed at 752.4 (CH_2_, CH_3_ twisting, rocking) and 758.2 (CH_2_ twisting) cm^–1^.^44,50^

For *n*-butane, two bands located at 958.7 (ν_16_, C–H rocking or twisting) and 2932.6 (ν_13_ CH_2_ *sym*. stretching)^54^ and three butene bands at C=C str. at 1642.8 cm^–1^, an H–C=C–H wag. vibration mode at 994.5 cm^–1^, and a vibration mode (=CH_2_ wag.) at 908.8 cm^–1^ are observed in this experiment. They were also observed by Kim et al.^54^

Four bands of naphthalene are observed at 1023.7 cm^–1^ (C–H bending), 1596.3 cm^–1^ (C–H bending or C–H out-of-plane), 1732.8 cm^–1^ (C–H bending), and 1960.3 cm^–1^ (C–H bending or ring deformation),^58^ as shown in Figure 3b,c. Four benzene bands are observed: two fundamental bands at 908.8 cm^–1^ (ν_18_ ring deformation or C–H bend) and 1037.5 cm^–1^ (ν_18_ C–H in-plane bending), one combination band at 1960.3 cm^–1^ (ν_17_ + ν_5_ C–H bend and ring deformation),^10,56^ and a C–H out-of-plane at 752.4 cm^–1^. Details are presented in Figure 3a,c,f. Only one ethane band is identified, a fundamental 2973.8 cm^–1^ (ν_10_ C–H stretching).^54,64^ In the current experiment, three bands are attributed to C_4_H_4_; the vinylacetylene that is thermodynamically less stable than the vinylacetylene methylenecyclopene isomer is visible through the ν_1_ C–H stretching, ν_6_ + ν_7_, and ν_6_ at 3288.1, 2973.8, and 1596.3 cm^–1^, respectively.^54,60^ Only one 2-methyl-1,3-butadiene or isoprene molecule is identified: 954.2 cm^–1^ as CH_2_ wagging/CH_2_ twisting.^48^

Several products are oxygenated molecules: carbon dioxide (CO_2_), identified by three bands at 655.5 cm^–1^ (ν_2_ CO_2_ bending), 2344.5 cm^–1^ (ν_3_ CO_2_ stretching), and 3609.2 cm^–1^ (2ν_2_ + ν_3_ CO_2_ bending);^43,47,49,62^ carbon monoxide, identified by two bands at 2137.2 cm^–1^ (ν_1_^12^CO str.)^43,49^ and 2091.6 cm^–1^ (ν_1_ stretching),^43,47,63^ corresponding to ^13^CO. Two acetaldehydes, CH_3_CHO, 758.2 cm^–1^ (ν_14_ CH bending) and ν_4_ CO stretching at 1722.8 cm^–1^,^53^ and a methanol band at 1032.3 cm^–1^ (ν_6_ CO stretching)^53,61^ are also observed. A band of ethylene oxides was also observed at ν_5_ 868.0 cm^–1^ ring deformation,^49^ with a vinyl alcohol, CH_2_CHOH, at 1642.8 cm^–1^ (ν_5_ CC stretching).^53^ Bands attributed to complex molecules are observed in the region of 1050–1080 cm^–1^ related to ethanol, CH_3_CH_2_OH, at 1093 cm^–1^;^55^ a band of HOCH_2_CH_2_OH, ethylene glycol, at 1090.1 cm^–1^,^57^ and a band at 1067.1 cm^–1^ that can be related to HCOCH_2_OH, glycolaldehyde,^61^ or HOCH_2_CH_2_OH, ethylene glycol.^57^

Bennett et al.^53^ performed laboratory studies to elucidate synthetic pathways for the formation of three C_2_H_4_O isomers—acetaldehyde (CH_3_CHO), ethylene oxide (C_2_H_4_O), and vinyl alcohol (CH_2_CHOH)—and demonstrated the relevance of these molecules for the astrochemical evolution of the interstellar medium.

Acetaldehyde and ethylene oxide have been proposed to play a significant role in amino acid synthesis^67^ and early metabolic pathways,^68^ respectively. Furthermore, the existence of ethylene oxide indicates the potential presence of a larger ring structure, furan (C_4_H_4_O), which is closely related to the sugars ribose and deoxyribose; these molecules, interconnected by phosphate(s), constitute the structural backbone of RNA and DNA, respectively.^69^

Figure 5 shows a comprehensive comparison of all molecular species formed during the irradiation of α-pinene and H_2_O ices by heavy ions as a function of ion fluence. The column densities of the identified products span several orders of magnitude, reflecting the complexity of the radiation-induced chemical network. Most species display a rapid increase in abundance at low fluences, followed by a plateau or saturation trend, suggesting a fluence-dependent formation limited by precursor depletion or destruction at higher doses.

**Figure 4:**
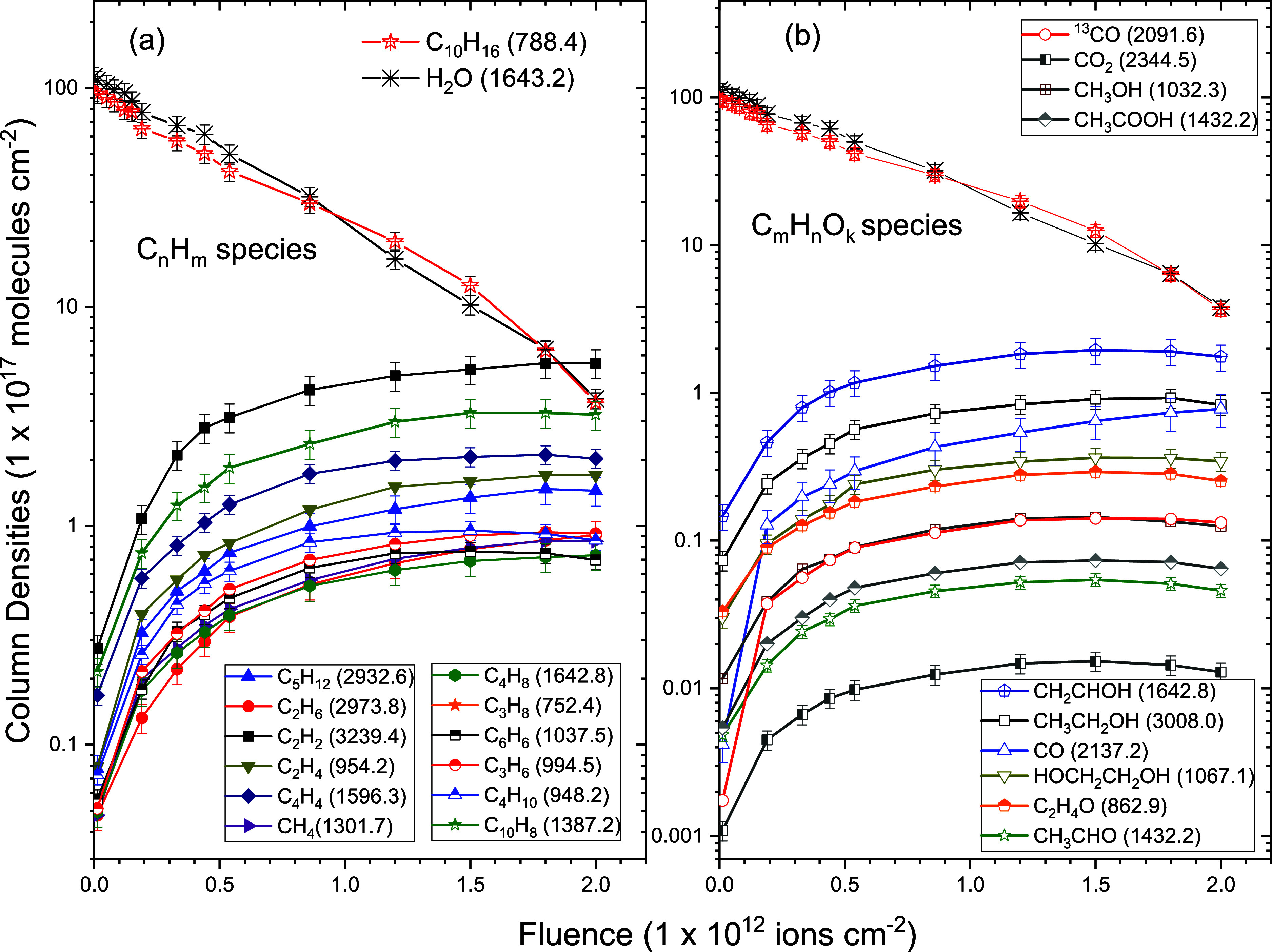
Column density (*N*(*F*)) evolution as a function of ion fluence of precursors: C_10_H_16_ and H_2_O and their product species created during radiolysis in (a) C_*m*_H_*n*_ species and (b) C_*m*_H_*n*_O_*k*_ species. Band wavenumbers in cm^–1^ that were used to estimate column densities for different molecules are shown in parentheses.

**Figure 5:**
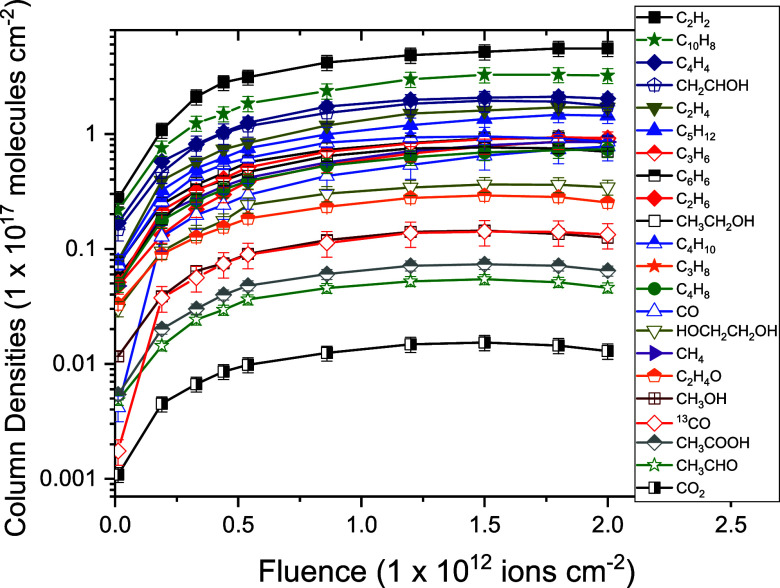
Evolution of the column densities of the detected molecular products as a function of ion fluence during the irradiation of α-pinene/H_2_O ice. The data include hydrocarbons, oxygenated organics, and small molecules such as CO and CO_2_. The figure highlights the relative abundance and formation behavior of each species. Error bars represent experimental uncertainties.

### Cross-Section Determination

When a molecular sample is exposed to an ion beam, the quantity of molecules in the initial target (precursor) is altered primarily by two mechanisms: radiolysis and sputtering. This means that modifications in the ice are caused by (i) the desorption of molecules from the ice surface and (ii) the formation of new molecules. The main information extracted from ion irradiation as a function of projectile fluence using infrared spectroscopy as an analytical technique is (i) the destruction cross-section (σ_d_) of the precursor molecular species, (ii) the formation cross-section (σ_f_) of new molecular species (products), and (iii) the desorption yield (*Y*).

### The α-Pinene and H_2_O Destruction Cross-Sections

During radiolysis, the column density (*N*) of the α-pinene molecules decreases. Since the ice samples used in the current measurements are thin compared to the penetration depth of Kr ions, the destruction cross-section is constant along the projectile track, and the column density evolution can be described by eq 2.

2 $$\frac{dN}{dF}=-\sigma_{d}N-Y\left(F\right)$$

where *N*(*F*) is the precursor column density at fluence *F* and *Y*(*F*) is the sputtering yield, both generally decreasing as the beam fluence increases. This equation, under certain realistic conditions, has been solved by Mejía et al.^70^ The solution of eq 2 is^70^

3 $$N\left(F\right)=\left(N_{0}-Y_{0}F\right)\exp\left(-\sigma_{d}F\right)$$

where *N*(0) is the precursor initial column density, *Y*_0_ is the initial sputtering yield, without the influence of the radiolysis, and σ_d_ is the destruction cross-section of precursors.

The evolution of the 788.4 cm^–1^ band has been used for the α-pinene destruction cross-section determination.^23^ For H_2_O, the column density was determined from the evolution of the absorbance of the 1643.2 cm^–1^ band. The cross-sections were determined from fittings using eq 3 shown in Figure 6. For pure α-pinene irradiated with a 61.3 MeV ^84^Kr^15+^ ion beam, the radiolysis cross-section obtained is σ_d_ = 5.4 × 10^–13^ cm^2^.^23^ Adding water to the mixture, the new cross-section is reduced to σ_d_ = 3.5 × 10^–13^ cm^2^ for α-pinene, while σ_d_ for water, it is 6.4 × 10^–13^ cm^2^; the sputtering yields are *Y*_0_ = 3.7 × 10^6^ molecules per impact for α-pinene and *Y*_0_ = 3.5 × 10^6^ molecules per impact for water.

**Figure 6:**
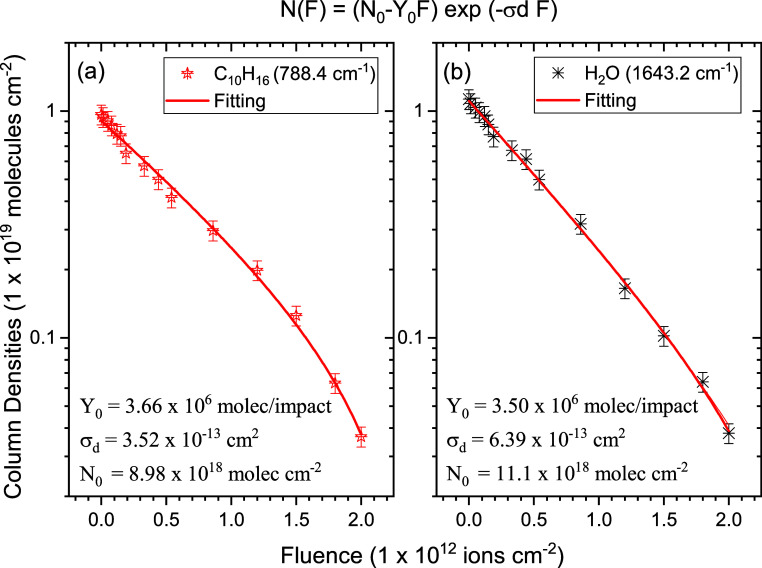
Column density (*N*(*F*)) evolution as a function of ion beam fluence for (a) α-pinene and (b) H_2_O precursors. The fittings are performed using eq 3 to determine the destruction cross-sections.^70^

### The Formation and Destruction Cross-Sections of Products

The products are classified into (i) fragments if they originate from a single precursor and (ii) hybrids (in the current experiment, hydrocarbons) if they are formed from two or more precursors. For (i), the evolution of the column density as a function of the beam fluence, observed for each fragment, follows the approximate expression used previously in the case of α-pinene.^44^

4 $$\frac{N_{k}\left(F\right)}{N_{0}}\approx \sigma_{f,k}\left[F-\frac{\left(\sigma_{d}+\sigma_{d,k}\right)}{2}F^{2}\right]$$

where σ_f,k_ is the apparent formation cross-section, the relevant parameter in this analysis, σ_d_ refers to the destruction of its precursor, and σ_d,k_ refers to the apparent destruction of the k product.^44^ Since sputtering is negligible for products at the beginning of the experiment, the apparent formation cross-sections can be considered equivalent to the true (or standard) formation cross-sections. The cross-sections of products are different from each other and generate the small variations observed in the *N*_k_ (*F*) evolutions. The column densities of the hydrocarbons were determined using the *A*-values presented in Table 4. For hybrids, chemical reactions are involved, and cross-section calculations are more difficult.^71^ Taking advantage of the fact that for the current mixture the ratio is about 1:1, eq 4 has also been employed. Cross-sections are expected to be correct within a factor of 2.

**Table 4:** Wavelengths, *A*-Values, and Destruction (σ_d,k_) and Formation (σ_f,k_) Cross-Sections of the Radiolysis Products^a^

Molecules Species	Position (cm^–1^)	*A*-value 10^–18^ cm molec^–1^	σ_f,k_	σ_d,k_
	Hydrocarbons	C_*n*_H_*m*_	10^–14^ cm^2^	10^–14^ cm^2^
CH_4_	1301.7	7.76 ^44,47^	0.98	1.4
C_2_H_2_	3239.4	5.0 ^44,47,49^	7.2	2.4
C_2_H_4_	954.2	15 ^44^	2.0	1.5
C_2_H_6_	2973.8	14.8 ^44^	0.86	1.7
C_3_H_6_	994.5	2.4 ^50^	1.2	1.1
C_3_H_8_	752.4	0.8 ^44,50^	1.1	2.3
C_4_H_4_	1596.3	1.4 ^72^	2.9	1.2
C_4_H_8_	908.8	6.0 ^54^	2.2	1.9
C_4_H_10_	958.7	1.8 ^72^	1.5	2.4
C_5_H_12_	2932.6	0.33 ^59^	1.7	1.8
C_6_H_6_	1037.5	2.5 ^56^	0.9	2.9
C_10_H_8_	1387.2	0.81 ^58^	4.2	1.7

Molecules Species	Position cm^–1^	*A*-value 10^–18^ cm molec^–1^	σ_f,k_	σ_d,k_
	Oxygenated products	C_*n*_H_*m*_O_*k*_	10^–14^ cm^2^	10^–13^ cm^2^
CO	2137.2	11 ^43,47^	0.65	2.9
^13^CO	2091.6	13.2 ^43,47,73^	0.21	1.1
CO_2_	2344.5	76 ^47,73^	0.02	5.5
CH_3_OH	1032.3	18 ^51,61^	0.21	3.9
CH_3_CHO	1432.2	3.7 ^53^	0.08	5.4
CH_3_COOH	1722.8	51.9 ^57^	0.12	5.2
C_2_H_4_O	862.9	11 ^53^	0.44	3.1
CH_2_CHOH	1642.8	0.51 ^53^	2.8	0.66
CH_3_CH_2_OH	3008.0	6.7 ^51^	0.13	1.1
HOCH_2_CH_2_OH	1067.1	0.07 ^51^	1.6	0.36

a The cross-sections were obtained by fitting the product’s column density evolutions with eq 4, with the errors varying between 5% and 15%. Absolute cross-sections of the oxygenated products have larger errors.

The column density evolutions of the observed hydrocarbons are presented in Figure 7. Figure 8 shows the column densities of the oxygenated products formed from radiolysis in a mixture of α-pinene and water molecules. Table 4 presents the *A*-values, the formation cross-sections (σ_f,k_), and the effective destruction cross-sections (σ_d,k_) of various hydrocarbons (C_*n*_H_*m*_) and oxygen-bearing molecules (C_*n*_H_*m*_O_*k*_).

**Figure 7:**
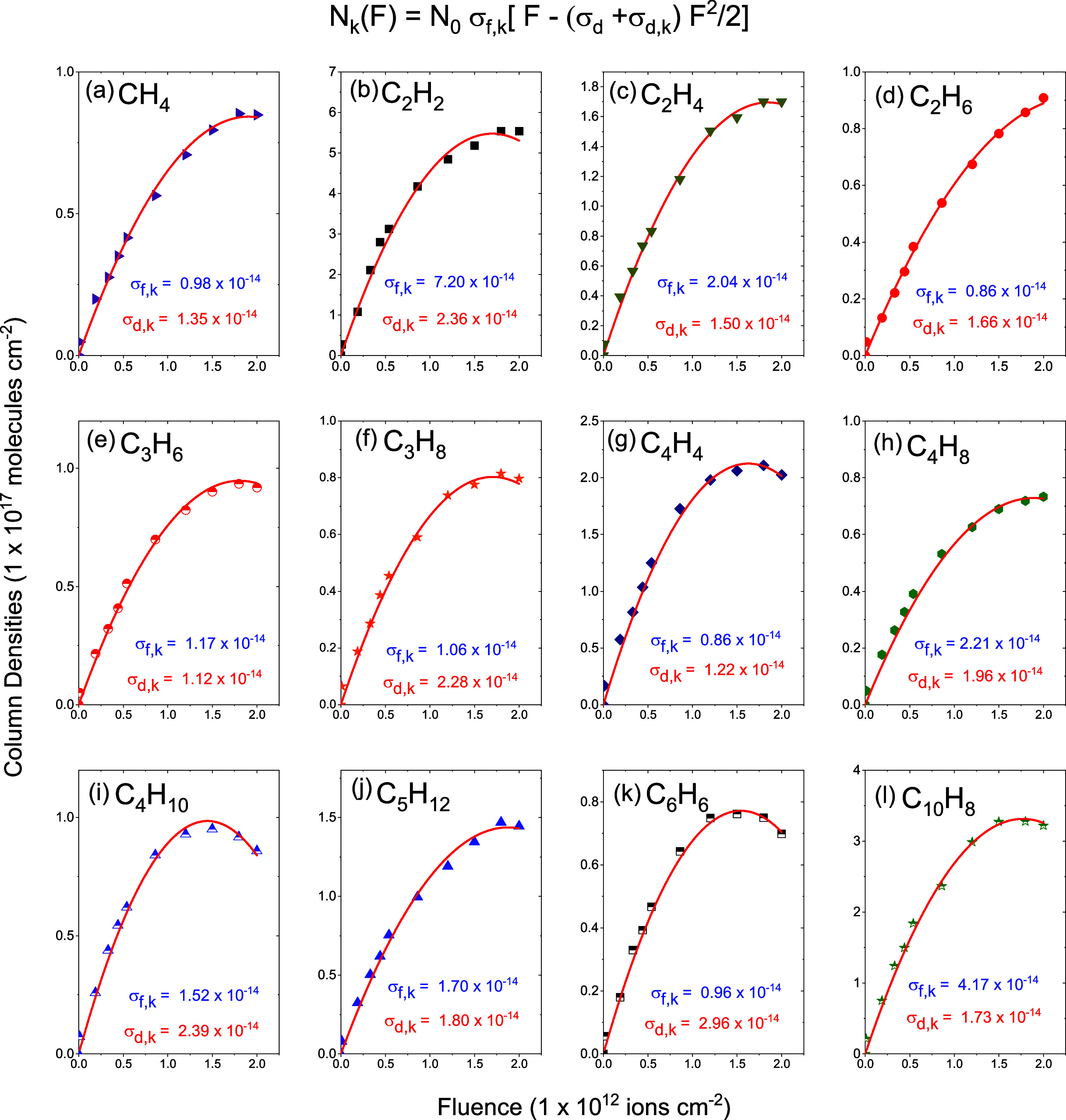
Column density evolution as a function of ion beam fluence for C_*m*_H_*n*_ products produced during radiolysis. Solid curves in red are fittings performed with eq 4 for (a) methane (CH_4_), (b) acetylene (C_2_H_2_), (c) ethylene (C_2_H_4_), (d) ethane (C_2_H_6_), (e) propylene (C_3_H_6_), (f) propane (C_3_H_8_), (g) vinylacetylene (C_4_H_4_), (h) butene (C_4_H_8_), (i) *n*-butane (C_4_H_10_), (j) pentane (C_5_H_12_), (k) benzene (C_6_H_6_), and (l) naphthalene (C_10_H_8_). It is important to note that the destruction cross-section for the formed hydrocarbon species was determined using only the α-pinene destruction cross-section value of 3.5 × 10^–13^ cm^2^.

**Figure 8:**
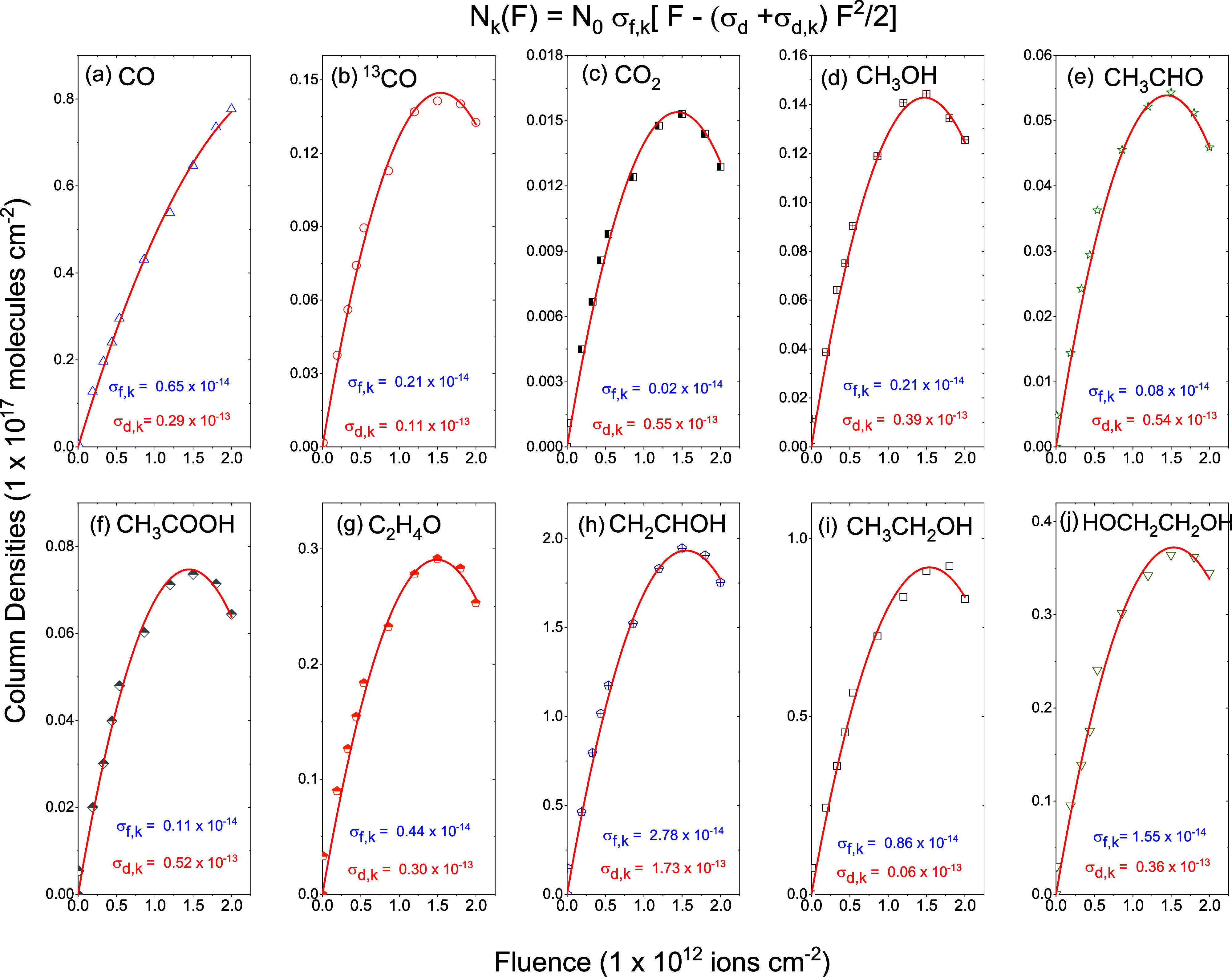
Column density evolution as a function of ion beam fluence for C_*n*_H_*m*_O_*k*_ products formed during radiolysis. Solid curves in red are fittings performed using eq 4. It is important to note that the destruction cross-section for the formed oxygenated species was determined using the H_2_O destruction cross-section value of 6.4 × 10^–13^ cm^2^. Absolute cross-sections have large errors because they depend on the mixture ratio of precursors.

Figures 7 and 8 show the column density fittings of the product species using eq 4. Column densities *N*_k_ (*F*) are characterized by (i) a quasi-linear increase at low fluence with a slope proportional to the formation cross-section, (ii) a maximum around *F* ≈ 10^12^ molecules cm^–2^, and (iii) a decrease of *N*_k_ for higher fluences. This evolution is due to the sum of the precursor and product destruction cross-sections. Figure 4 shows that the destruction cross-sections for the precursor bands are about 10^–13^ cm^2^, while the average value of the formation cross-sections (Table 4) for its produced species (hydrocarbons and oxygenated molecules) is about 10^–14^ cm^2^.

The dependence of the formation and destruction cross-sections on the molecular structure provides insight into the susceptibility to radiolysis and the molecular stability of products, summarized in Figure 9 for the C_*n*_H_*m*_ products. We considered the two types of processed samples: pure α-pinene (ref de Barros et al. (2024))^23^ and the α-pinene mixture with water (data presented in Table 4).

**Figure 9:**
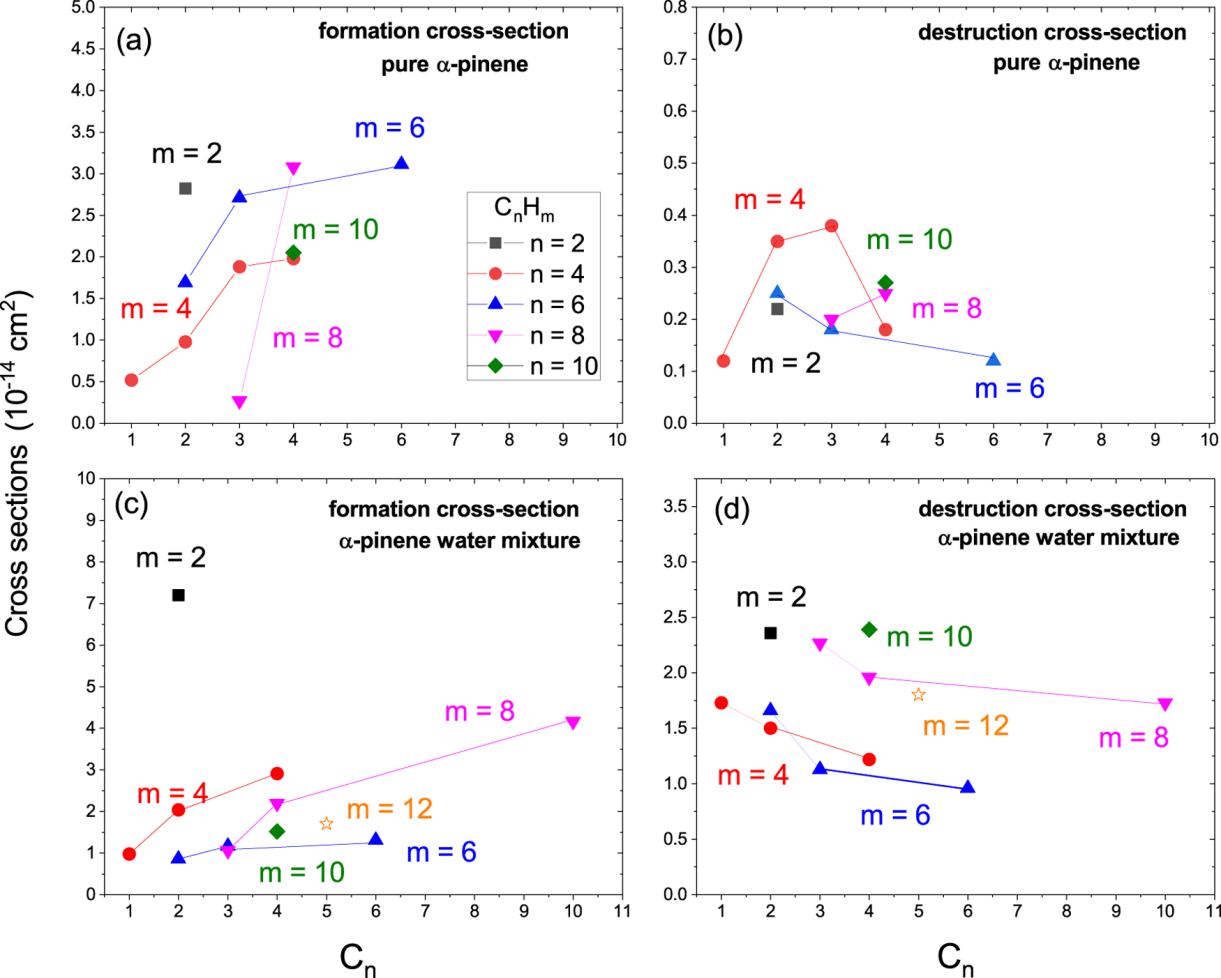
Dependence of the formation (left) and destruction (right) cross-sections on the number of carbon atoms (*n*) for the hydrocarbon products C_*n*_H_*m*_. The irradiated samples are pure pinene (top) and the α-pinene–water mixture (bottom). Note the σ_f,k_ increasing and the σ_d,k_ decreasing dependence as a function of carbon atoms (C_*n*_).

### Discussion on the Formation Cross-Sections of Products

The C_*n*_H_*m*_ formation cross-sections (σ_f,k_), presented in Figure 9a,c as a function of *n*, highlight the efficiency of molecular synthesis under the experimental conditions given for pure α-pinene and for the α-pinene–water mixture, respectively. The main trends are as follows

(i) The formation cross-section increases as n increases for both the pure and mixture samples.
(ii) Concerning the C_*n*_H_2_ products, just the first member (C_2_H_2_) is observable.
(iii) The presence of water does indeed change significantly the evolution cross-sections of the C_*n*_H_4_ series but not of the C_*n*_H_6_ series.
(iv) The presence of water alters the slope of the C_*n*_H_8_ series. Specifically, it increases considerably the formation cross-section of C_3_H_8_, reduces and flattens the C_4_H_8_, and enables the detection of C_10_H_8_.
(v) C_2_H_2_ (acetylene) has the highest formation cross-section for the mixture with water and for pure α-pinene.

Among the hydrocarbons, C_2_H_2_ (acetylene) has the highest formation cross-section: 7.2 × 10^–14^ cm^2^. The high σ_f,k_ value of acetylene may be attributed to its relatively simple molecular structure, allowing for straightforward formation pathways from smaller radicals and fragments during hydrocarbon processing. In addition, acetylene is a fundamental intermediate species in many chemical reactions, which contributes to its significant formation rate. The molecular stability of the C_*n*_H_2_ series has recently been discussed by Díaz Soto et al.^74^ For the most stable isomers, they found that the C_2_H_2_, C_4_H_2_, C_5_H_2_^+^, and C_6_H_2_ series have a linear structure.

C_4_H_4_ exhibits the relatively high formation cross-section for the C_*n*_H_4_ series at 2.9 × 10^–14^ cm^2^, while CH_4_ and C_2_H_6_ have the lowest values of around 0.98 × 10^–14^ cm^2^ and 0.86 × 10^–14^ cm^2^, respectively. Ethane may not form as easily because it must undergo hydrogenation steps to form from smaller hydrocarbons, a process that is not expected to occur as frequently in this experiment. For pure and mixture samples, methane has the lowest formation cross-section compared with other hydrocarbons. This can be explained by several factors:

(a) methane consists of four strong CH bonds (bond energy ∼ 439 kJ/mol), making it highly stable.^75^ This stability means that the formation of methane from precursor radicals or smaller hydrocarbons is less favorable than the formation of less saturated molecules;
(b) unlike unsaturated hydrocarbons such as acetylene (C_2_H_2_) or benzene (C_6_H_6_), methane does not have resonance stabilization, which could make its formation energetically more challenging;
(c) methane is often a final product of hydrocarbon decomposition, rather than an intermediate in their formation. This property means that under conditions where hydrocarbons are forming, methane may not be the primary product, and
(d) CH_3_ radicals, a key precursor to methane, can also lead to larger hydrocarbon formation rather than methane itself. If these radicals encounter other hydrocarbon fragments, they can form ethane (C_2_H_6_) or more complex species instead of methane.

### Discussion on the Destruction Cross-Sections

The lines joining the C_*n*_H_*m*_ destruction cross-sections (σ_d,k_) values, presented in Figure 9b,d, describe the dependence on *n* for pure α-pinene and for the α-pinene–water mixture, respectively. The major trends pointed out are as follows:

(i) The destruction cross-section generally decreases as *n* increases; this behavior is the same for the pure and mixture samples.
(ii) The exception is the C_*n*_H_4_ series for pure pinene, which presents a maximum for C_*n*_H_3_ and the lowest value for CH_4_. Such CH_4_ disappearance in water solution is attributed to the reactions CH_4_ + H_2_O → CO + 3H_2_ and CH_4_ + H_2_O → CO_2_ + H_2_ that should occur in the hot projectile track; indeed, CO and CO_2_ are products observed in the spectra of the irradiated sample with water.
(iii) The presence of water generally increases the cross-sections of C_*n*_H_*m*_ series. Then oxygen is available to produce oxygenated molecules, which reduces the densities of hydrocarbons.
(iv) Formation and destruction cross-sections have the opposite trends with respect to the molecular size: while the former increases, the latter decreases with *n*. Higher production followed by lower destruction means that synthesis of large species is favored relative to small ones. The ability to produce larger species during radiolysis is most likely due to the carbon atom’s multiple linkage properties.
(v) Roughly, the destruction cross-sections are 1 order of magnitude lower than the formation ones. This guarantees that the abundance of products increases at the beginning of irradiation. After a certain fluence, the precursor’s densities decrease, obliging the product’s production rate to diminish. Moreover, since the products’ densities are still increasing, their destruction rates also increase, forcing their leveling off. This general behavior is seen in Figures 7 and 8.

The size and structure of the molecules enhance the resilience against radiation-induced destruction. Saturated hydrocarbons tend to be more susceptible to destruction than unsaturated hydrocarbons. Indeed, C_2_H_2_ (acetylene, σ_d,k_ = 2.4 × 10^–15^ cm^2^) is more prone to radiolysis than C_2_H_6_ (ethane, σ_d,k_ = 1.7 × 10^–15^ cm^2^), likely due to weaker single bonds in alkanes compared to stronger triple bonds in alkynes.

Aromatic hydrocarbons (e.g., benzene and naphthalene) are more resistant to radiolysis than smaller aliphatic hydrocarbons. Benzene (C_6_H_6_) has σ_d,k_ = 2.9 × 10^–15^ cm^2^, whereas naphthalene (C_10_H_8_) has an even lower σ_d,k_ = 1.7 × 10^–15^ cm^2^. This trend can be attributed to the fact that larger molecules generally possess more extensive delocalized electron systems and multiple bonding sites, which enhance their structural stability and resistance to radiolytic fragmentation compared to that of smaller, more saturated hydrocarbons.

For the oxygenated molecules, CH_2_CHOH (vinyl alcohol) has the highest formation cross-section at σ_d,k_ = 2.8 × 10^–13^ cm^2^, while CO_2_ has one of the lowest at 0.2 × 10^–14^ cm^2^. Vinyl alcohol is an unstable intermediate in some chemical reactions and does not exist freely in nature for long periods. It quickly tautomerizes (rearranges) into acetaldehyde (CH_3_CHO), which is more stable. The high formation cross-section of vinyl alcohol may be attributed to its synthesis via multiple precursor pathways, involving both hydrocarbons and oxygen-bearing species, whereas CO_2_ is typically formed by oxidation reactions that may be less efficient under the experimental conditions.

Overall, the trend in formation cross-sections suggests that larger, more complex molecules benefit from cumulative reaction pathways, while smaller species form with lower efficiency due to either fewer reaction intermediates or competitive destruction processes.

For instance, light hydrocarbons tend to exhibit larger destruction cross-sections. CH_4_ (methane) has a destruction cross-section of σ_d,k_ = 1.4 × 10^–14^ cm^2^, which is comparable to that of the heavier molecule C_10_H_8_ (naphthalene), with σ_d,k_ = 1.7 × 10^–14^ cm^2^. This result suggests that in this case, molecular size and structure do not significantly influence the resistance to radiation-induced destruction.

In the case of oxygen-bearing molecules (C_*n*_H_*m*_O_*k*_), the oxygenated species generally exhibit destruction cross-sections higher than that of hydrocarbons of similar size, indicating their greater susceptibility to radiation-induced breakdown. For example, carbon dioxide (CO_2_) has some of the highest σ_d,k_ values, with 5.5 × 10^–13^ cm^2^, and so does carbon monoxide (CO) ∼ 3.0 × 10^–13^ cm^2^. This indicates that simple carbon–oxygen bonds are more prone to dissociation. Larger organic molecules with oxygen (e.g., CH_3_OH, CH_3_CHO) display moderate destruction cross-sections, indicating that functional groups influence stability. Methanol (CH_3_OH) has a σ_d,k_ of ∼ 3.9 × 10^–13^ cm^2^, significantly higher than that of ethanol (CH_3_CH_2_OH), for which σ_d,k_ = 1.1 × 10^–13^ cm^2^, suggesting that additional carbon in ethanol increases resilience.

In a comparison of hydrocarbons and oxygenated products, it can be observed that oxygenated molecules generally have destruction cross-sections that are higher than those of pure hydrocarbons, probably because of weaker carbon–oxygen bonds. This is especially evident in CO_2_, which has the highest values, σ_d,k_, followed by acetaldehyde (CH_3_CHO) and methyl formate (CH_3_COOH). For hydrocarbons, increasing the molecular mass generally reduces their stability σ_d,k_, but for oxygen-containing molecules, this trend is less pronounced, showing that functional groups play an important role in stability.

Smaller hydrocarbons exhibit a higher volatility σ_d,k_, making them more prone to destruction. Aromatic and larger hydrocarbons are more stable than smaller aliphatic species. Oxygen-bearing molecules have significantly higher destruction cross-sections than hydrocarbons, highlighting their vulnerability to radiolysis. Carbon oxides (CO, CO_2_) and alcohols (CH_3_OH) show the highest destruction cross-sections, pointing out their instability under irradiation. These findings are relevant for understanding molecular evolution in astrophysical environments, where radiolysis plays a key role in chemical transformations.

For the oxygenated molecules, those that are formed from both precursors, a similar behavior on the molecular size is observed (see Table 4): small products have relatively low and high formation and destruction cross-sections (e.g., for CO, they are 0.65 and 29 × 10^–14^ cm^2^, and even CO_2_ has 0.02 and 55 × 10^–14^ cm^2^, respectively), while for large products, they are larger and lower, respectively (e.g., for CH_2_CHOH, they are around 2.8 and 6.6 × 10^–14^ cm^2^ and for ethylene glycol (HOCH_2_CH_2_OH), they are 1.6 and 3.6 × 10^–14^ cm^2^, respectively).

### Comparison of the Radiolysis of α-Pinene in Water Ice and Pure α-Pinene

In our previous study (de Barros et al., 2024 ^23^), we irradiated pure α-pinene ice with the same ion beam (^84^Kr^15+^) at 61.3 MeV, using an identical experimental setup. That work allowed us to establish the primary fragmentation and rearrangement pathways of α-pinene under heavy-ion bombardment in the absence of water. Among the identified products in the pure α-pinene study were unsaturated hydrocarbons such as ethylene (C_2_H_4_), propene (C_3_H_6_), 1-butene (C_4_H_8_), benzene (C_6_H_6_), and naphthalene (C_10_H_8_), formed through ring opening, dehydrogenation, and carbon–carbon bond rearrangements. In the present study, we observed new species not detected in the pure α-pinene case, most notably small oxygenated molecules such as vinyl alcohol (CH_2_CHOH), acetaldehyde (CH_3_CHO), and other partially oxidized hydrocarbons. The presence of water enables additional radiolytic mechanisms, including hydroxylation and hydration reactions, that are inaccessible in the absence of H_2_O. Therefore, we attribute the appearance of these oxygen-bearing species to secondary reactions between radiolysis products (e.g., radicals or unsaturated fragments) and water-derived species such as OH and H. This comparison clearly supports the conclusion that water ice plays an active chemical role in the radiolysis of α-pinene, not merely as an inert matrix. It alters the product distribution and reaction pathways, demonstrating that part of the observed molecular complexity arises from interactions between the hydrocarbon precursor and the radiolysis fragments of H_2_O. These findings are consistent with earlier studies of hydrocarbon–water mixtures under ionizing radiation (e.g., Moore & Hudson, 1998;^1^ Bennett et al., 2005 ^53^) and underscore the importance of considering mixed ices when simulating astrochemical processes on icy bodies.

## Astrophysical Implications

While α-pinene has not yet been directly detected on Titan, it is a representative biogenic monoterpene with a chiral structure and a reactive hydrocarbon backbone that mimics the behavior of complex organic aerosols in Titan’s atmosphere. Titan’s upper atmosphere is rich in hydrocarbons such as methane, ethane, propane, benzene, and various nitriles, many of which are formed through photochemistry and cosmic ray interactions. The formation of aromatic and unsaturated compounds observed in our experiment, such as benzene, acetylene, and naphthalene, parallels Titan’s observed chemical inventory, suggesting similar radiation-induced pathways could occur under Titan-like conditions.

Moreover, water ice is abundant on the surface of icy moons like Europa and Enceladus and may also exist beneath Titan’s surface. Although Titan’s surface is largely composed of hydrocarbons and tholins, interactions between organic molecules and subsurface water ice could occur via cryovolcanic or impact processes. In this sense, this α-pinene–water ice mixture serves as a simplified analog for potential organic-ice interfaces in Titan’s subsurface environment or in icy grains in its atmosphere.^76–78^

The use of heavy-ion irradiation in our study mimics the high-energy cosmic rays that penetrate deep into planetary surfaces and atmospheres, including those of Titan, Europa, and Enceladus. These galactic cosmic rays (GCRs), composed mainly of high-energy protons and heavy nuclei, are capable of driving chemical transformations in subsurface ices and organic-rich layers on icy moons and other solar system bodies.^79,80^ While the precise temperatures, ice composition, and radiation flux vary across these environments, Titan being rich in hydrocarbons and nitriles, Europa dominated by water ice with potential surface oxidants, and Enceladus exhibiting active cryovolcanism, the experimental approach isolates the fundamental radiolytic chemistry relevant to all three settings.^81,82^ Therefore, the results presented here offer insight into how complex organic molecules may evolve in extraterrestrial environments where water ice coexists with hydrocarbons and is continuously processed by energetic particle irradiation.^1,83^

The radiolysis of α-pinene and water by Kr beams highlights the role of ionizing radiation in the transformation of organic molecules in icy environments.^17,78,84^ We need to know about these processes to understand the chemical complexity seen in the interstellar medium (ISM) and on icy bodies in the Solar System, where cosmic rays and ion irradiation have a big impact on the molecular inventory. The findings also advance our understanding of prebiotic chemistry and the potential habitability of extraterrestrial environments.^85–87^

Upon irradiation of the C_10_H_16_/H_2_O (1:1) ice mixture, 12 hydrocarbons were formed, belonging to the class of complex organic molecules (COMs), which are of significant interest in laboratory astrophysics.^10,58,60^ Among these, benzene stands out due to its relevance as a building block for polycyclic aromatic hydrocarbons (PAHs) and its confirmed presence on Titan, where it contributes to this Saturn’s moon’s rich organic chemistry.^86–89^ Other hydrocarbon isomers, such as *n*-butane, butene, and propylene, were also identified. Furthermore, several oxygen-bearing COMs of astrobiological significance were detected, including isomers of acetaldehyde, vinyl alcohol, ethylene oxide, glycolaldehyde, methyl formate, and acetic acid. These species have been reported in hot molecular cores and may act as key intermediates in the formation of prebiotic molecules.^25,53,57,61,90^

Ionizing radiation reacts with α-pinene and water to form many complex organic molecules. This process is similar to what is thought to happen in interstellar ice, icy moons, and other places beyond our solar system. Heavy-ion irradiation induces radiolysis, breaking molecular bonds and generating reactive species such as radicals, ions, and smaller molecules, which recombine to form new compounds.^23,91^ This radiolysis process contributes to the synthesis of prebiotic molecules, including alcohols, aldehydes, ketones, and hydrocarbons, which are of particular interest in the study of the origins of life.^87,88,92,93^

From an astrochemical and planetary perspective, the present experiment simulates conditions found in icy bodies exposed to cosmic rays or heavy ions, such as those in the Kuiper Belt, in Europa, or in the Titan atmosphere. The insights gained from these studies help elucidate the chemical evolution of organic matter under extreme radiation environments and its potential role in planetary habitability.^72,76,86,94^

## Remarks and Conclusions

This study provides insight into the radiation-induced chemistry of hydrocarbon-rich ice layers on Saturn’s moon Titan and water-rich ice on Enceladus. On Jupiter’s moon Europa, where water ice coexists with potential organic contaminants, radiolysis may drive similar pathways, contributing to the observed surface features and potential subsurface chemistry.^10,44,70,76^

The radiolysis of α-pinene and water by Kr beams points out the role of ionizing radiation in the transformation of organic molecules in icy environments. These processes are crucial for understanding the chemical complexity observed in the ISM and on icy solar system bodies, where cosmic rays and ion irradiation play a pivotal role in shaping the molecular inventory. Such findings also improve our understanding of prebiotic chemistry and the potential habitability of extraterrestrial environments.^52,78,94^

The present study investigated the radiolysis of α-pinene (C_10_H_16_) mixed with water ice (H_2_O) in a 1:1 ratio under cosmic ray-like conditions. The destruction cross-section of α-pinene in the mixture was determined to be ∼3.5 × 10^–13^ cm^2^, lower than the value for pure α-pinene (∼5.4 × 10^–13^ cm^2^), indicating a stabilizing effect of water. The sputtering yields were also quantified: a yield of ∼3.7 × 10^6^ molecules per ion for α-pinene and 3.5 × 10^6^ molecules per ion for water.

Twelve hydrocarbons (C_*n*_H_*m*_) and 10 oxygenated products (C_*n*_H_*m*_O_*k*_) formed due to irradiation were observed. The key hydrocarbon products included acetylene (C_2_H_2_), ethylene (C_2_H_4_), benzene (C_6_H_6_), naphthalene (C_10_H_8_), and propane (C_3_H_8_). Among oxygenated species, the most abundant species were glycolaldehyde (HCOCH_2_OH), vinyl alcohol (CH_2_CHOH), ethanol (CH_3_CH_2_OH), and methyl formate (CH_3_OCHO). The cross-section values for the formation of the products varied, with the highest at ∼7.2 × 10^–14^ cm^2^ for C_2_H_2_, indicating that these molecules readily form under irradiation. However, C_6_H_6_ (benzene) has a lower formation cross-section, indicating less efficient formation, but it has a higher destruction cross-section, implying that it is more easily fragmented once it is formed. Oxygenated molecules such as CO_2_, in particular, have the highest destruction cross-section, while CH_3_COOH (acetic acid) and CH_3_CHO (acetaldehyde) also exhibit high destruction rates (∼5.0 × 10^–13^ cm^2^), making them more susceptible to fragmentation under irradiation. In contrast, ethylene glycol (HOCH_2_CH_2_OH) and CH_2_CHOH (vinyl alcohol) show lower destruction cross-sections (∼0.5 × 10^–13^ cm^2^), indicating that they are more stable under irradiation than their oxygenated counterparts. When water was added to the mixture, it changed the reaction pathways, and more oxygenated products were formed than with pure α-pinene.^8,85^

The destruction cross-section (σ_d,k_) generally decreases with increasing molecular mass, making larger hydrocarbons more resistant to radiolysis. Aromatic compounds, such as benzene and naphthalene, exhibit a lower stability σ_d,k_ than smaller aliphatic hydrocarbons, indicating greater stability. Oxygen-bearing molecules tend to have higher destruction cross-sections than hydrocarbons, with CO and CO_2_ being the most radiation-sensitive. Functional groups significantly influence the molecular resilience, as seen in alcohols and aldehydes. These trends provide insights into molecular stability in astrophysical environments affected by radiation.

Oxygenated molecules had higher destruction cross-sections than hydrocarbons. CO, CO_2_, CH_3_OH, and CH_3_CH_2_OH exhibited significant degradation rates, indicating that oxygen-bearing species are more sensitive to radiolysis. Comparison with pure ice irradiation showed a higher stability of certain organic products in the presence of water. The presence of CH_3_OH and CH_3_CHO, which are crucial intermediates in the synthesis of amino acids and sugars, clearly shows the potential of interstellar ice chemistry to contribute to the inventory of prebiotic molecules delivered to planetary systems.

The interaction of ionizing radiation with the α-pinene and water mixture leads to the formation of various complex organic molecules, similar to processes in interstellar ice and icy moons. Aromatic compounds, such as benzene and naphthalene, display higher stability than smaller aliphatic hydrocarbons. Functional groups significantly influence molecular resilience, with alcohols and aldehydes forming as key radiolysis products. These findings provide valuable insights into molecular transformations in astrophysical environments and their implications for prebiotic chemistry.

In summary, this study provides critical insights into the formation and stability of complex organic molecules (COMs) under interstellar conditions. The results show how water influences the radiolysis of α-pinene, leading to the formation of oxygen-rich species. These findings contribute to astrochemical models by improving predictions of organic molecule evolution in icy cosmic environments.
